# Nail clubbing in laxative abuse: case report and review of the literature

**DOI:** 10.1186/s40337-019-0236-4

**Published:** 2019-03-05

**Authors:** Olivia A. Charlton, Philippa Dickison, Saxon D. Smith, Simon D. Roger

**Affiliations:** 1 Department of Dermatology, Royal North Shore Hospital, Sydney, 0419125257 Australia; 2 The Dermatology and Skin Cancer Centre, Gosford, Australia; 3 Department of Renal medicine, Gosford Hospital, Gosford, Australia

**Keywords:** Eating disorder, Clubbing, Nail clubbing, Laxative abuse

## Abstract

**Background:**

The link between clubbing and laxative abuse has been reported several times in the literature, in all cases in young females. The nature of this relationship is not understood.

**Case:**

A young female, with no history of hepatic, pulmonary or malignant disease was found to have nail clubbing in the context of laxative abuse. A literature review revealed several similar cases.

**Conclusion:**

Laxative abuse is an important consideration in the assessment of clubbing in populations at risk of eating disorders, to prevent over-investigation and facilitate management of the eating disorder itself. This case highlights a new clinical presentation of an eating disorder.

**Case:**

A 36-year-old woman was being reviewed by a renal specialist for renal impairment and electrolyte disturbances, in the context of a background of multiple renal calculi 4 years prior, hypokalaemia and hypercalcaemia. The attending nephrologist brought attention to her nails, which demonstrated clubbing. She stated that she had had clubbing for 10 years, and that it was of gradual onset and not associated with any pain. There was no history of hepatic, cardoipulmonary or malignant disease.

The patient also had a background of gastrointestinal reflux disease, depression, anxiety and an eating disorder. Her current medications included: mirtazapine, olanzapine, quetiapine, temazepam, antacid, pantoprazole and potassium supplements. She was also taking at least 15 tablets of either senna or docusate sodium (Coloxyl) and senna on a daily basis. She further reported taking 90 to 100 senna tablets at the peak of her eating disorder 4 years prior, at which point she weight 28 kg.

On examination, the patient was found to have bilateral clubbing of all finger nails (Fig. 1). She was underweight, weighing 41.7 kg; her body mass index (BMI) was 16.8. The rest of the examination was normal. Investigations demonstrated chronic renal disease (creatinine 130 μmol/L), hypercalaemia (2.8 mmol/L), hypokalaemia (3.1 mmol/L) and low vitamin D (12 nmol/L). Her renal biopsy demonstrated a significantly thickened glomerular basement membrane and acute tubular injury, however there was no clear diagnosis. A computed tomography (CT) scan did not reveal any underlying malignancy, and an echocardiogram did not indicate a cardiac cause for the nail clubbing.

**Fig. 1:**
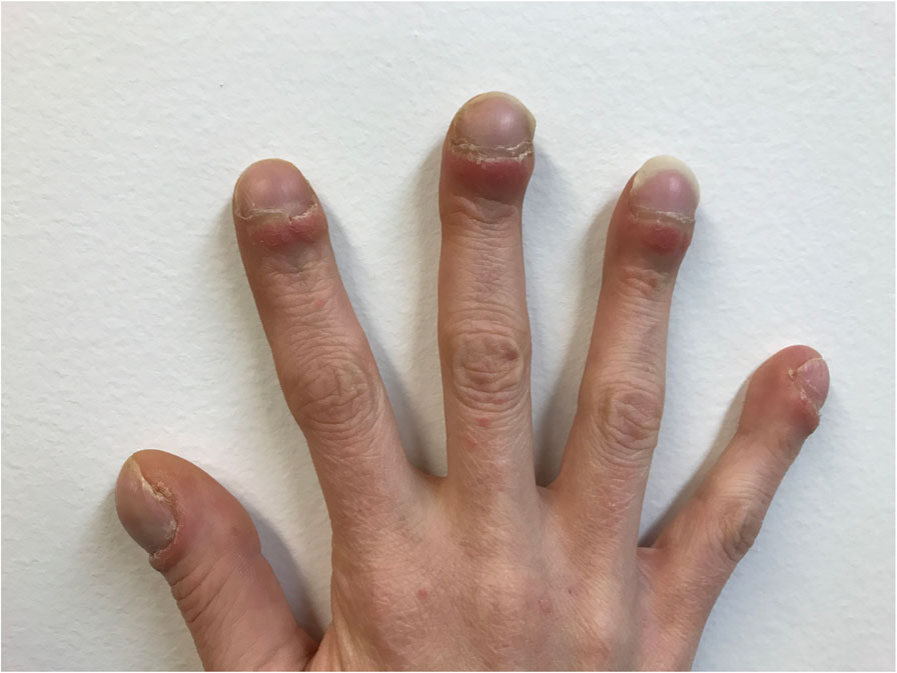
The tips of the fingers are enlarged, and the nails curve around the fingertips

The absence of symptoms, previous history, and investigation results indicated that the most likely cause of her finger clubbing was her use of senna. Unfortunately this patient has continued daily use of laxatives, in spite of repeated recommendations that she cease using them, and the clubbing has not resolved.

## Discussion and conclusions

Finger clubbing has been associated with cardiac, pulmonary, neoplastic and gastrointestinal diseases or infections [1]. It is also part of the syndrome of primary or secondary hypertrophic osteoarthropathy (HOA). HOA is characterised by periostosis of long bones, joint pain and clubbing [1].

The pathophysiology underlying the development of clubbing is not entirely understood. The proposed mechanisms relate to altered vascular dynamics and vasodilation in the fingers; increased production of plasma growth hormone; and increased concentration of megakaryocytes impacting in the fingertip circulation [1]. None of the theories explaining the development of clubbing encompasses all associations.

An example of an unexplained association with clubbing is laxative abuse. This link has been reported several times in the literature since Silk et al. [2] in 1975 (Table 1). Like this case, all the cases were females who admitted to consuming high amounts of senna tablets to control their weight. In 3 cases, the link was established or confirmed after senna derivatives were identified in the urine [2–4]. Similarly, many of the previously reported cases had associated hypokalaemia, chronic renal failure or fluid retention. Additionally, one report also linked a patient’s hypercalcaemia to the calcium in senna tablets (12.5 mg calcium per senna tablet) [5].

**Table 1:** Case reports which have identified clubbing in patients consuming laxatives

Manuscript	Patient details	Associated	Laxative	FU
Silk et al. (1975) [2]	26yo F	History of persisting vomiting Normal UECs	100–200 Senna tablets/day	Resolved with cessation of aperients
Prior and White (1978) [4]	24yo F	Hyperventilation leading to tetany Hypokalaemia	~ 50 Senna tablets/day	Continued laxative use and clubbing
Malmquist et al. (1980) [8]	30yo F 48 kg	Aspartylglucosaminuria Radius bones curved Fluctuating fluid retention	Pursennid (Senna)	Continued laxative use and clubbing
Levine et al. (1981) [6]	65yo F 24 kg	Hypokalaemia Hypoalbuminaemia	Senna	Resolution with cessation of senna
Armstrong et al. (1981) [9]	21yo F	HOA	> 3 Senna tablets/day	Resolution within 6 months of ceasing senna
Fitzgerald & Redmond (1983) [7]	44yo F	Hypokalaemia Paranoid schizophrenia	4–40 Senna tablets/day	Changed to Bisacodyl; clubbing “regressing”
Pines et al. (1983) [10]	28yo F 43 kg	Hypokalaemia	20–30 Senna tablets/day	Continued clubbing and laxative use
	35yo F 46 kg	IDDM Smoker	Paraffin oil Senna	Continued clubbing and laxative use
Currie et al. (2007) [3]	62yo F	Mild chronic renal failure Hypokalaemia	Senna Lactulose	Continued clubbing and laxative use
Lim, Hooke and Kerr (2008) [5]	36yo F BMI 17.7	Smoker Hypercalcaemia Hypokalaemia Fluid retention HOA*	50–100 Senna tablets/day	Persistent despite reported reduction in Senna

*This diagnosis was challenged in a Letter to the editor [11]; HOA - Hypertrophic osteoarthropathy

The link between laxative abuse, specifically senna, and clubbing is unknown. It has been postulated to be related to diarrhoea or malnutrition [6, 7]. Importantly, the diagnosis can only be established after pulmonary, cardiac, neoplastic and gastrointestinal causes are excluded. Interestingly the clubbing was reported to resolve upon cessation of senna [2, 8, 9].

## Conclusion

This case adds to the increasing, though small, number of reports linking senna and finger clubbing. It is important to consider laxative abuse in cases of clubbing, especially in young, underweight female patients. Awareness of this association may prevent over-investigation and facilitate the diagnosis and management of an eating disorder.

## Abbreviations

BMI: Body mass index
CT: Computed tomography
HOA: Hypertrophic osteoarthropathy
